# Porcine reproductive and respiratory syndrome prevalence and processing fluids use for diagnosis in United States breeding herds

**DOI:** 10.3389/fvets.2022.953918

**Published:** 2022-11-24

**Authors:** Mariana Kikuti, Carles Vilalta, Juan Sanhueza, Claudio Marcello Melini, Cesar A. Corzo

**Affiliations:** ^1^Department of Veterinary Population Medicine, University of Minnesota, Saint Paul, MN, United States; ^2^Unitat mixta d'Investigació IRTA-UAB en Sanitat Animal, Centre de Recerca en Sanitat Animal (CReSA), Campus de la Universitat Autònoma de Barcelona (UAB), Barcelona, Spain; ^3^Instituto de Investigación y Tecnología Agroalimentaria (IRTA), Programa de Sanitat Animal, Centre de Recerca en Sanitat Animal (CReSA), Campus de la Universitat Autònoma de Barcelona (UAB), Barcelona, Spain; ^4^Departamento de Ciencias Veterinarias y Salud Pública, Facultad de Recursos Naturales, Universidad Católica de Temuco, Temuco, Chile

**Keywords:** disease outbreak, porcine reproductive and respiratory syndrome, epidemiology, swine diseases, epidemiological monitoring

## Abstract

**Introduction:**

Processing fluids have been recently adopted by the U.S. swine industry as a breeding herd PRRS monitoring tool due to their increased representativeness of animals within the herd. Here, we use the Morrison Swine Health Monitoring Project (MSHMP) database, representative of ~50% of the U.S. swine breeding herd, to describe processing fluids submissions for PRRS diagnosis and their relation to PRRS prevalence and time to stability over time between 2009 and 2020.

**Methods:**

An ecological time series Poisson regression modeling the number of status 1 farms and weekly percentage of processing fluids submissions for PRRS diagnosis was done. Time to stability was calculated for sites that detected a PRRS outbreak within the study period and modeled through a proportional hazards mixed effect survival model using production system as a random-effect factor and epiweek as a panel variable.

**Results:**

Processing fluids diagnosis submissions increased starting in 2017. The difference between each year's highest and lowest weekly prevalence averaged 10.9% between 2009 and 2017, whereas it averaged 5.0% in 2018–2020 period. Each year's lowest weekly prevalence ranged from 11.3 to 19.5% in 2009–2017 and from 22.4 to 29.2% in 2018–2020. We also detected an increasing proportion of breeding sites that did not reach stability within 1 year of reporting an outbreak (chi-square for trend *p* < 0.0001). The total time to stability was not associated with the region of the country in which the site was located, the site’s air filtration status, its PRRS status before the outbreak, or the different statuses a site achieved to be classified as stable, when accounting for the production system in the multivariate model. However, a higher proportion of system-wide processing fluids use was associated with increased time to stability.

**Discussion:**

Altogether, the temporal concurrence of processing fluids used for PRRS virus monitoring suggests that the adoption of this sampling strategy may help explain the changes observed in PRRS status 1 prevalence since 2018, although further studies are still needed.

## Introduction

Porcine reproductive and respiratory syndrome (PRRS), caused by *Betaarterivirus suid 1* (PRRSV-1) and *Betaarterivirus suid 2* (PRRSV-2) (1), has been a burden on the U.S. swine industry for over three decades, causing a major economic impact (2). Efforts have been made in the last decade to share data and conduct analyses of different levels to control, prevent, and eliminate it (3). Herd surveillance of PRRS is a crucial activity to understand the dynamics of transmission, but also to control and eliminate the virus from a production system. For breeding herds, a standardized terminology was proposed based on serum samples of wean-age pigs (4). Blood collection from 30 to 60 randomly selected due-to-wean piglets for reverse transcriptase polymerase chain reaction (RT-PCR) testing was the industry standard up until recently to detect the presence of the virus, assuming this age group accurately represented PRRS status in the breeding herd (4). Since then, new monitoring strategies have arisen, such as the use of processing fluids to screen the newborn pig population in breeding herds, leading to an updated classification system including this new sampling methodology (5).

Processing fluids are the serosanguineous exudate obtained from tails and testicles as part of castration and tail-docking practices. This sampling methodology has become widely adopted in the U.S. swine industry since it is convenient, easy to adapt to daily farm chores, reliable, and cost-effective, and the sample can be collected from a large number of piglets to monitor for PRRS in breeding herds (6, 7). This sample type is potentially more sensitive in detecting the PRRS virus when only a few animals are infected. The increased sensitivity to detect if PRRS is present in a given farm when using processing fluids may rely on the increased representativeness, given this sample is generated through routine practices in a large number of 3–5 day-old pigs. The number of litters that can be aggregated to reduce testing costs depends on the cycle threshold (Ct) value of the positive sample being diluted. The lower the Ct-value (i.e., the higher the viremia in the individual animals), the higher the level of aggregation that can be achieved. An aggregated sample of 40 litters is predicted to detect a PRRS-virus positive litter when the Ct-value of that positive sample is below 30 (8). Testing aggregated processing fluid samples reduces diagnosis cost while optimizing labor compared to blood collection of 30–60 due-to-wean piglets (7). Because more animals are being represented on the farm's monitoring program, this strategy is potentially more sensitive in detecting PRRS, particularly at lower within-farm prevalence.

PRRS prevalence in breeding herds, or the proportion of the breeding herds that are classified as PRRS-positive and weaning PRRS-virus-positive piglets at a given point in time, is a product of both the incidence (number of new herds that detect a new PRRS strain) and the time that a herd requires to control the within-herd transmission of a wild-type or vaccine strain. This is commonly known as time to stability. Once the herd starts to consistently produce PRRS-negative pigs (i.e., there is an absence of clinical signs and a lack of detectable viremia in weaning-age pigs for at least 90 days), the herd is known to have reached stability (4). Many veterinarians and producers also opt to pursue elimination (i.e., negative PRRS shedding and exposure status), in which case reaching stability can be one of the steps toward elimination. The adoption of more sensitive monitoring tools, such as the molecular testing (e.g., RT-PCR) of processing fluid samples for PRRS-virus RNA detection, can potentially increase our ability to detect disease occurrence. Thus, we aimed to describe the temporal correlation between the use of processing fluids for PRRS virus detection and the overall PRRS prevalence in the U.S. breeding herd population, as well as the average time it took farms to reach stability over the studied period.

## Methods

An ecological study was designed to investigate the association between PRRS prevalence in breeding herds and the use of processing fluids for PRRS diagnosis. The studied population was all the production systems participating in the Morrison Swine Health Monitoring Project (MSHMP), which represents ~50% of the U.S. swine-breeding herd (3). The MSHMP collects data generated by participating systems' routine monitoring for PRRS and other infectious diseases. For PRRS, participating systems report changes (i.e., outbreak, stability, elimination) in the health status for each of their breeding farms weekly according to the American Association of Swine Veterinarian PRRS breeding herd classification guidelines proposed in 2011 (4) for the duration of this study. Briefly, status 1 indicates herds that are positive unstable in which within-herd viral transmission and shedding is present. Status 2 represents seropositive breeding herds classified as positive stable with a sustained lack of viremia in weanling pigs. Statuses 3 and 4 are herds classified as provisional negative or negative, where no viral shedding exists but seropositive (in the case of status 3) or seronegative (in the case of status 4) animals are present at the farm. Farms in status 2 were further classified into status 2fvi (field virus inoculation) or 2vx (modified-live virus vaccine use) according to the elimination or control strategy adopted. Therefore, the weekly PRRS prevalence, defined as the percentage of farms in each status category from July 2009 to December 2020, was calculated on a weekly basis according to participant reports and was plotted over time.

Most (75%, 30/40) participating companies conduct their PRRS-virus diagnosis for at least a fraction of their sites in two main Veterinary Diagnostic Laboratories (VDLs), University of Minnesota (UMN) and Iowa State University (ISU). Data regarding the sample type submitted for PRRS-virus diagnosis by RT-PCR were obtained for all submissions from MSHMP participants between January 2015 and December 2020 from both UMN and ISU VDLs. Data from both VDLs were combined, and the percentage of sample types submitted monthly throughout the studied period was plotted. Additionally, an ecological time series Poisson regression modeling the number of status 1 farms and the weekly percentage of processing fluids submissions for PRRS diagnosis was done (9) using the number of total sites enrolled in MSHMP that were sharing weekly health statuses as exposure. A second model added a flexible spline function to account for seasonality and long-term trends in the status 1 prevalence with 52 knots. Both models were compared based on Akaike information criterion (AIC) and the best model's deviance residuals were plotted over the studied time period.

Time to stability was calculated for sites that detected a PRRS outbreak within the study period. It comprised the number of weeks from when the breeding herd status was changed to status 1 for the first time because of an outbreak to when it was reclassified into status 2, 2fvi, 2vx indicating it had reached a stable status. Time to stability for farms that had a PRRS outbreak (i.e., sites that transitioned to status 1) during 2015-2016 (before processing fluids testing was available) were compared to those in which the outbreak occurred during 2018–2020 (after processing fluids testing was available) using the Wilcoxon rank–sum test. The percentage of farms that did not reach stability within 1 year according to the year in which the PRRS outbreak was detected was compared using chi-square for trend. Additionally, each farm's time to stability from farms that experienced a PRRS outbreak from January 2015 to December 2020 (a time in which PCR data was also available) was modeled through a proportional hazards mixed effect survival model using production system as a random-effect factor and epiweek as a panel variable according to: (1) the region of the country, as defined by the Swine Health Information Center (SHIC) (10); (2) air filtration status; and (3) system-wide processing fluids use, defined by the overall proportion of samples submitted for PRRS diagnosis that were processing fluids per production system per year. Sites' contribution times were censored in December 31, 2020 for sites that were still unstable. Factors associated with time to stability with a *p* ≤ 0.10 in the bivariate model were included in the full multivariate model. A stepwise backwards elimination process was done using *p* ≤ 0.05 as a cutoff to define the final model. All statistical analysis was conducted using STATA 17.0 (11).

## Results

**Figure 1:**
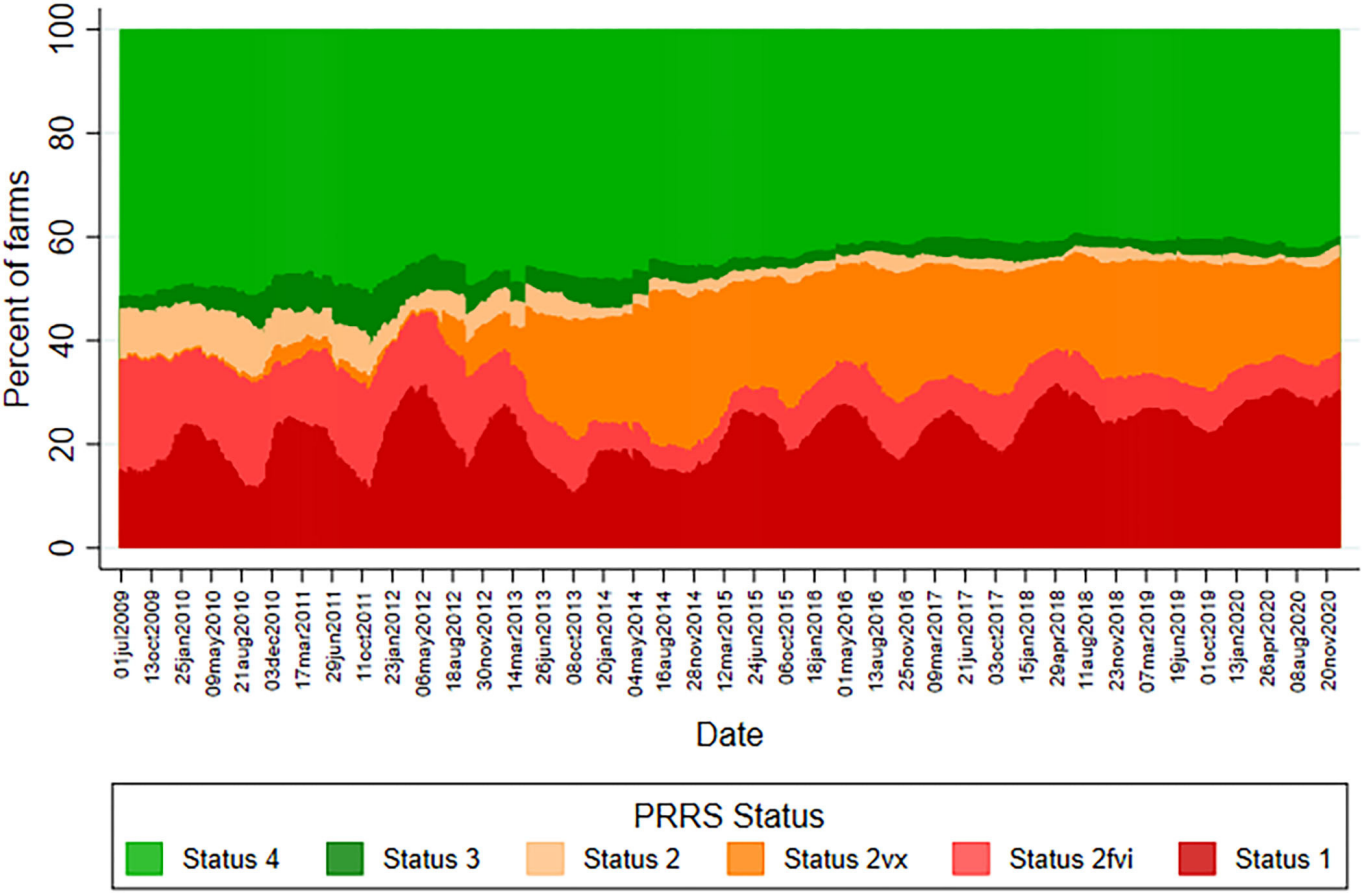
PRRS prevalence of United States sow herd status from 1,140 herds participating in the Morrison Swine Health Monitoring Project beginning July 01, 2009.

A median of 889 sites were followed weekly throughout the study period (a minimum of 364 sites and a maximum of 1,051 sites). The lowest median weekly number of sites that contributed was 365 in 2009 and the highest was 1,033 in 2019. A decrease in PRRS statuses 2 and 2fvi weekly prevalence has been observed since 2013, accompanied by an increase in status 2vx prevalence (Figure 1). This change in the pattern regarding status 2 and its sub-classifications remained practically unchanged for the remainder of the studied period. Regarding the weekly prevalence of status 1 sites, a seasonal pattern of higher prevalence in winter months going into spring and lower prevalence in summer and fall months from 2009 to late 2013 was observed (Supplementary Table 1). Maximum and minimum weekly prevalence for each year during this period ranged from 19.2 to 31.5% and 10.7 to 15.6%, respectively. The percentage difference between the highest and lowest weekly prevalence in each year was 5.9% in 2009, in which monitoring started in the middle of the year, and ranged from 12.9 to 17.1% for 2010–2013 with a median yearly difference of 14.1% throughout this period. In 2014, however, status 1 weekly prevalence showed less pronounced peaks and valleys compared to the observed in previous years, as shown by the 5.0% difference between the highest (19.2%) and lowest (14.2%) weekly prevalence for the year. For 2015–2017, the median difference between the yearly highest and lowest weekly prevalence increased to 10.6%, ranging from 8.4 to 10.9%, illustrating that peaks and valleys returned to being more clearly defined throughout the period. However, since 2018, the seasonality of status 1 prevalence has not been as clearly defined as in previous years. Not only did the median yearly difference between the higher and lower weekly prevalence decrease to 5.0%, but 2018–2020 were also the years with the highest lowest weekly prevalence. The lowest weekly prevalence observed in 2018, 2019, and 2020 was 23.8, 22.1, and 26.3%, respectively.

**Figure 2:**
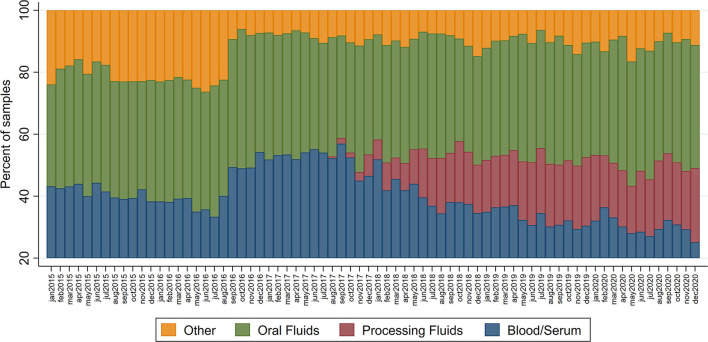
Percentage of sample type specimens submitted for PRRS RT-PCR diagnosis at the UMN and ISU Veterinary Diagnostic laboratories amongst MSHMP participants.

The median monthly PRRS RT-PCR submissions during the study period was 7,936, ranging from 6,815 to 9,397. Based on this dataset, processing fluids specimens started being submitted in August 2017 and became frequently used during 2018–2020, comprising 15.2 to 26.5% of all diagnostic specimen submissions for that period (Figure 2). Since August 2018, a median of 1,544 (ranging from 1,106 to 1,958) processing fluids samples were submitted monthly for PRRS RT-PCR detection.

Prevalence of status 1 farms over time, as well as the weekly percentage of processing fluids submissions over time, are plotted in Supplementary Figure 1. The naïve Poisson model to assess status 1 sites and the percentage of processing fluids use association without adjustment for long-term patterns showed an incidence rate ratio (IRR) of 1.009 (95% CI 1.007–1.01, *p* < 0.001), suggesting a slight increase in the number of status 1 farms when the percentage of processing fluid use is higher (AIC = 1,715.029). However, after adding the spline function to account for long-term trends, this association does not remain statistically significant (IRR = 0.999; 95% CI 0.998–1.001; *p* = 0.36) and the model is improved (AIC = 1,696.557). The deviance residual is shown in Supplementary Figure 2, which suggests that this model's performance was poorer earlier in the study period.

A total of 1,436 herds had a PRRS outbreak, of which 1,203 reached stability within the studied period. The median time to stability was lower in sites that detected the PRRS outbreak between 2015 and 2016 [median 32 weeks; interquartile range (IQR): 19–48], before processing fluids started being used, than between 2017 and 2020 (median 35 weeks; IQR: 22–56; *p* = 0.002), after they were adopted. Similarly, the chi-square for trend for the proportion of sites that did not reach stability within 1 year was significant (*p*=0.01). Of all sites that detected a PRRS outbreak in 2015, 21.7% did not reach stability within 1 year. This percentage decreased to 17.8% in 2016, and then increased to 23.5, 31.4, 28.4, and 27.7% in 2017, 2018, 2019, and 2020, respectively.

Factors associated with each farm's time to stability are described in Table 1. In the bivariate analysis, region, system-wide processing fluids use, and status when stable were associated with time to stability at *p* ≤ 0.10 and selected for inclusion in the multivariate model. In the multivariate model, however, only processing fluids use remained associated with time to stability. The higher the system-wide use of processing fluids in the year the outbreak was detected, the lower the rate to reach stability (i.e., higher time to stability) was found. Contrasts of the global mean of time to stability by each level of categorical variables were also assessed (Supplementary Table 2); however, no difference between each level and the global mean was observed.

**Table 1:** Bivariate proportional hazards mixed effect survival model results between time to stability and investigated predictors, using production system as a random-effect factor.

	***n***	**Bivariate**		
		**HR**	**95% CI**	***p*-value**
**Region**
2	283	1	–	–
3	682	0.71	0.50–1.01	0.06
4	38	0.53	0.27–1.05	0.07
5	359	1.06	0.66–1.72	0.80
**Air filtration**
None	479	1	–	–
Partial	33	0.87	0.52–1.46	0.61
Year-Round	180	1.24	0.94–1.62	0.13
Processing fluids use^*^	1,137	0.16	0.08–0.34	<0.001
**Status when stable**
2	170	1	–	–
2fvi	460	1.43	1.09–1.86	0.01
2vx	573	1.10	0.89–1.35	0.38
**Previous status**
2fvi	378	1.22	0.99–1.51	0.07
3	40	1.15	0.79–1.68	0.47
4	210	1.04	0.85–1.28	0.70
2vx	750	1	–	–

CI, confidence interval.

* Production system-wide proportion of submitted samples that were processing fluids in the year the PRRS outbreak was detected.

## Discussion

Given that the PRRS yearly incidence was lower from July 2018 to June 2020 (average cumulative incidence of 24.2%) than from July 2014 to June 2018 (average cumulative incidence of 29.9%) (12), and that overall positivity in PRRS RT-PCR submissions was within the expected range (13), a reasonable hypothesis for the increase in prevalence of status 1 farms during 2018 onwards is that farms that had PRRS outbreaks are remaining in status 1 for longer periods of time than in previous years. This is supported by the PRRS prevalence pattern observed, in which the cyclical decrease in prevalence is no longer clearly observed during the summer and fall seasons, and by the significant chi-square for trend in the percentage of sites that do not reach stability within 1 year. However, these are crude estimates with no adjustments made for production system or other factors that could potentially influence disease reporting.

The temporal concurrence of processing fluids used for PRRS virus monitoring suggests that the adoption of this monitoring strategy may help to explain the changes observed in PRRS status 1 prevalence since 2018. This is supported by the positive association found between a higher system-wide proportion of processing fluids submitted for PRRS diagnosis and a higher time to stability. An important limitation of this analysis was that we were not able to discern what laboratory tests were responsible for each site's status change, and whether the farms with a higher time to stability were the ones using processing fluids. Instead, we used system-wide processing fluids use as a proxy. However, we hypothesize that it is possible that farms at low within-herd prevalence were being misclassified as stable (e.g., false stability) prior to the adoption of a more sensitive sampling strategy. Nonetheless, this temporal correlation does not necessarily mean causation, and other factors might also be involved in this change in prevalence pattern. Particularly since while the prevalence of status 1 farms plateaued from late 2019, the frequency of processing fluids use did not significantly change from mid-2018. Additionally, it is important to note that due-to-wean piglets might yield positive PRRS RT-PCR results even after two consecutive negative 4-week batches processing fluids results, thus monitoring should not rely solely on this monitoring strategy (14).

Other factors must be considered as additional factors explaining the increased prevalence, particularly because PRRS occurrence and processing fluids use were not consistently associated throughout the different ecological analysis. For example, the introduction of new strains in already infected herds will contribute to them remaining in category 1. Specific virus strains may have developed the capacity to persist for longer periods of time at the population level, making the time to stability longer than expected. Viral strains associated with PRRS outbreaks were not assessed in this study. The new L1C 144 PRRS variant (15) only emerged as a significant health problem in late 2020 and remains restricted to one region of the U.S. Thus, we expect its effect in PRRS prevalence trends would be minimal in this study. However, the countrywide outbreaks of porcine epidemic diarrhea virus (PED) in 2013 and 2014 (16, 17) could have affected PRRS detection, since all efforts were focused on the detection, control, and elimination of this new disease that heavily affected the U.S. swine industry. There are ~40% of the breeding herds in category 3–4 (18), which is certainly a good indicator of practitioners and producers continuing to work toward maintaining naïve herds and working toward a naïve status. Additionally, the adoption of processing fluids and the decision to pursue elimination may vary widely according to the production system or perceived disease pressure within a given region. To account for this effect on the farms' time to stability, production system was added as a random effect in our model and region was assessed as a possible associated factor. Nevertheless, that might not have captured all the nuances regarding these factors that could affect time to stability.

Lastly, stability is considered to be a herd moving from status 1 into status 2, 2fvi, 2vx. However, for both statuses 2fvi (field-virus inoculation) and 2vx (modified-live virus vaccine usage), replacement gilts and in some cases sows and weanling pigs continue to be exposed to PRRS virus intentionally. A previous study demonstrated that sites that used modified-live vaccine as an outbreak intervention strategy achieved stability later than the ones that used field-virus inoculation (19). However, although we did not assess the role of interventions during the unstable period, sites that were classified as 2fvi had a higher rate in reaching stability than sites that were classified as 2 in the bivariate analysis, but that association was not sustained in the multivariate model. Additionally, a previous study with a similar dataset showed a shorter time to stability in farms that experienced PRRS outbreaks from a previous status 2fvi, 3, or 4 compared to sites previously at status 2vx (20). Here, previous status 2fvi was only marginally associated with a higher rate to reach stability compared to sites previously in status 2vx, but this was also not sustained in the multivariate model.

This study not only describes the industry-wide adoption of processing fluids as a monitoring tool for PRRS diagnosis, but also a temporal correlation between its adoption and an increased PRRSV prevalence and time to stability in recent years. Despite this, additional studies are necessary to ascertain the direct relationship between processing fluids use and longer time to stability.

## Data availability statement

The datasets presented in this article are not readily available because the data that support the findings of this study are generated and owned by the production systems participating in this study and is available on request from the corresponding author. The data are not publicly available due to privacy restrictions. Requests to access the datasets should be directed to corzo@umn.edu.

## Ethics statement

No ethical approval was required as this article describes data collected through routine veterinary diagnosis by the farms' veterinarians as part of standard care.

## Author contributions

MK and CAC contributed to the conception and design of the study. MK performed data analysis with critical contributions from CV, JS, and CMM. MK wrote the first draft of the manuscript. All authors contributed to manuscript revision, read, and approved the submitted version.

## Funding

The Morrison Swine Health Monitoring Project is a Swine Health Information Center (SHIC, www.swinehealth.org) funded project, Project # 20-172 SHIC (MK, CV, JS, CMM, and CAC). The funders had no role in study design, data collection and analysis, decision to publish, or preparation of the manuscript.

## Conflict of interest

The authors declare that the research was conducted in the absence of any commercial or financial relationships that could be construed as a potential conflict of interest.

## Publisher's note

All claims expressed in this article are solely those of the authors and do not necessarily represent those of their affiliated organizations, or those of the publisher, the editors and the reviewers. Any product that may be evaluated in this article, or claim that may be made by its manufacturer, is not guaranteed or endorsed by the publisher.

## Supplementary material

Supplementary figure 1 Weekly prevalence of status 1 farms and weekly percentage of processing fluids submissions over time.

Supplementary figure 2 Deviance residual of the Poisson model with adjustment for long-term patterns with spline function.

Supplementary Table 1 Lowest, median and highest weekly PRRS prevalence by year. ^*^2009 weekly prevalence comprise data from July to December only.

Supplementary Table 2 Contrasts of global mean of time to stability by each level of categorical variables.

## References

[1] Walker PJ Siddell SG Lefkowitz EJ Mushegian AR Adriaenssens EM Dempsey DM. Changes to virus taxonomy and the statutes ratified by the international committee on taxonomy of viruses 2020. Arch Virol. (2020) 165:2737–48. 10.1007/s00705-020-04752-x 32816125

[2] Holtkamp D Kliebenstein J Neumann E Zimmerman J Rotto H Yoder T. Assessment of the economic impact of porcine reproductive and respiratory syndrome virus on United States pork producers. J Swine Heal Prod. (2013) 21:72–84. 10.31274/ans_air-180814-2816121604

[3] Perez AM Linhares DCL Arruda AG VanderWaal K Machado G Vilalta C. Individual or common good? Voluntary data sharing to inform disease surveillance systems in food animals. Front Vet Sci. (2019) 6:194. 10.3389/fvets.2019.00194 31294036 PMC6598744

[4] Holtkamp DJ Polson DD Torremorell M Morrison B Classen DM Becton L. Terminology for classifying swine herds by porcine reproductive and respiratory syndrome virus status. J Swine Heal Prod. (2011) 19:44–56. Available online at: https://www.aasv.org/shap/issues/v19n1/v19n1p44.pdf 22138772

[5] Holtkamp D Torremorell M Corzo CA Linhares DCL Almeida MN Yeske P. Proposed modifications to porcine reproductive and respiratory syndrome virus herd classification. J Swine Heal Prod. (2021) 29:261–70. Available online at: https://www.aasv.org/shap/issues/v29n5/v29n5p261.html

[6] Vilalta C Sanhueza J Alvarez J Murray D Torremorell M Corzo C. Use of processing fluids and serum samples to characterize porcine reproductive and respiratory syndrome virus dynamics in 3 day-old pigs. Vet Microbiol. (2018) 225:149–56. 10.1016/j.vetmic.2018.09.006 30293648

[7] Lopez WA Angulo J Zimmerman J Linhares D. Porcine reproductive and respiratory syndrome monitoring in breeding herds using processing fluids. J Swine Heal Prod. (2018) 26:146–50. Available online at: https://www.aasv.org/shap/issues/v26n3/v26n3p146.html

[8] Vilalta C Baker J Sanhueza J Murray D Sponheim A Alvarez J. Effect of litter aggregation and pooling on detection of porcine reproductive and respiratory virus in piglet processing fluids. J Vet Diagnostic Investig. (2019) 31:625–8. 10.1177/1040638719852999 31122156 PMC6857019

[9] Bhaskaran K Gasparrini A Hajat S Smeeth L Armstrong B. Time series regression studies in environmental epidemiology. Int J Epidemiol. (2013) 42:1187–95. 10.1093/ije/dyt092 23760528 PMC3780998

[10] Swine Health Information Center. Rapid Response Program. (2022). Available online at: https://www.swinehealth.org/rapid-response-to-emerging-disease-program/ (accessed September 11, 2022).

[11] StataCorp. Stata Statistical Software: Release 17. StataCorp (2021).

[12] Morrison Swine Health Monitoring Project. PRRS Cumulative Incidence Beginning July 01, 2009. Morrison Swine Health Monitoring Project (2021). Available online at: https://vetmed.umn.edu/centers-programs/swine-program/outreach-leman-mshmp/mshmp/mshmp-prrs-figures

[13] Trevisan G Linhares DCL Magalhaes E Linhares L Crim B Dubey P. Swine Disease Reporting System: Report #40. Swine Health Information Center (2021). Available online at: https://www.swinehealth.org/domestic-disease-surveillance-reports/ (accessed June 2, 2021).

[14] Trevisan G Jablonski E Angulo J Lopez WA Linhares DCL. Use of processing fluid samples for longitudinal monitoring of PRRS virus in herds undergoing virus elimination. Porcine Health Manag. (2019) 5:18. 10.1186/s40813-019-0125-x 31388438 PMC6670174

[15] Kikuti M Paploski IAD Pamornchainavakul N Picasso-Risso C Schwartz M Yeske P. Emergence of a new lineage 1c variant of porcine reproductive and respiratory syndrome virus 2 in the United States. Front Vet Sci. (2021) 8:752938. 10.3389/fvets.2021.752938 34733906 PMC8558496

[16] American Association of Swine Veterinarians. UMN Case Report. American Association of Swine Veterinarians (2013). Available online at: https://www.aasv.org/aasv%20website/Resources/Diseases/PED/PEDVBiosecurity.pdf (accessed January 20, 2007).

[17] Morrison Swine Health Monitoring Project. PED EWMA analysis for years 2013–2021. Morrison Swine Health Monitoring Project (2022). Available online at: https://vetmed.umn.edu/centers-programs/swine-program/outreach-leman-mshmp/mshmp/ped-charts

[18] Morrison Swine Health Monitoring Project. PRRS prevalence of sow herd status beginning July 01, 2009. Morrison Swine Health Monitoring Project (2021). Available online at: https://vetmed.umn.edu/centers-programs/swine-program/outreach-leman-mshmp/mshmp/mshmp-prrs-figures

[19] Linhares DCL Cano JP Torremorell M Morrison RB. Comparison of time to PRRSv-stability and production losses between two exposure programs to control PRRSv in sow herds. Prev Vet Med. (2014) 116:111–9. 10.1016/j.prevetmed.2014.05.010 24931129

[20] Sanhueza J Vilalta C Corzo C Arruda A. Factors affecting porcine reproductive and respiratory syndrome virus time-to-stability in breeding herds in the midwestern United States. Transbound Emerg Dis. (2018) 66:823–30. 10.1111/tbed.13091 30520570

