# Supplementary material for: Porcine reproductive and respiratory syndrome prevalence and processing fluids use for diagnosis in United States breeding herds
Source: Front Vet Sci. 2022 Nov 24;9:953918. doi: 10.3389/fvets.2022.953918 (PMC9730796; doi:10.3389/fvets.2022.953918)
Supplement: Supplementary Table 1 — Lowest, median and highest weekly PRRS prevalence by year. *2009 weekly prevalence comprise data from July to December only. [file Table_1.docx]

**Supplementary Table 1.** Lowest, median and highest weekly PRRS prevalence by year.

| Weekly PRRS prevalence | 2009* | 2010 | 2011 | 2012 | 2013 | 2014 | 2015 | 2016 | 2017 | 2018 | 2019 | 2020 |
| --- | --- | --- | --- | --- | --- | --- | --- | --- | --- | --- | --- | --- |
| Lowest | 14.29% | 11.38% | 11.29% | 15.56% | 10.67% | 14.22% | 16.13% | 16.90% | 18.16% | 23.75% | 22.11% | 26.28% |
| Median | 15.38% | 19.14% | 20.70% | 24.69% | 16.40% | 16.65% | 22.10% | 23.45% | 22.67% | 27.78% | 25.48% | 28.98% |
| Highest | 20.22% | 24.32% | 25.41% | 31.49% | 27.75% | 19.17% | 26.79% | 27.79% | 26.48% | 31.77% | 27.10% | 30.81% |

*2009 weekly prevalence comprise data from July to December only.
