# Supplementary material for: Porcine reproductive and respiratory syndrome prevalence and processing fluids use for diagnosis in United States breeding herds
Source: Front Vet Sci. 2022 Nov 24;9:953918. doi: 10.3389/fvets.2022.953918 (PMC9730796; doi:10.3389/fvets.2022.953918)
Supplement: Supplementary Table 2 — Contrasts of global mean of time to stability by each level of categorical variables. [file Table_2.docx]

**Supplementary Table 2.** Contrasts of global mean of time to stability by each level of categorical variables.

|  | df | chi2 | P>chi2 | Sidak P>chi2 | Contrast | Delta-method std. err. | Sidak [95% conf. interval] | |
| --- | --- | --- | --- | --- | --- | --- | --- | --- |
| Region |  |  |  |  |  |  |  |  |
| (2 vs mean) | 1 | 2.98 | 0.08 | 0.30 | -15.38 | 8.91 | -37.58 | 6.82 |
| (3 vs mean) | 1 | 0.41 | 0.52 | 0.95 | 5.40 | 8.39 | -15.50 | 26.30 |
| (4 vs mean) | 1 | 1.43 | 0.23 | 0.65 | 18.88 | 15.77 | -20.41 | 58.16 |
| (5 vs mean) | 1 | 0.83 | 0.36 | 0.83 | -8.90 | 9.77 | -33.22 | 15.43 |
| Status when stable |  |  |  |  |  |  |  |  |
| (2 vs mean) | 1 | 2.73 | 0.10 | 0.27 | 6.28 | 3.80 | -2.79 | 15.36 |
| (2fvi vs mean) | 1 | 5.87 | 0.02 | 0.05 | -8.50 | 3.51 | -16.89 | -0.12 |
| (2vx vs mean) | 1 | 0.55 | 0.46 | 0.84 | 2.22 | 3.00 | -4.95 | 9.39 |
| Previous status |  |  |  |  |  |  |  |  |
| (2 vs mean) | 1 | 1.88 | 0.17 | 0.53 | 6.66 | 4.86 | -5.43 | 18.76 |
| (2fvi vs mean) | 1 | 1.21 | 0.27 | 0.72 | -6.76 | 6.14 | -22.06 | 8.53 |
| (3 vs mean) | 1 | 0.16 | 0.69 | 0.99 | -3.75 | 9.37 | -27.08 | 19.58 |
| (4 vs mean) | 1 | 0.42 | 0.52 | 0.95 | 3.85 | 5.95 | -10.97 | 18.67 |
| Air filtration |  |  |  |  |  |  |  |  |
| (None vs mean) | 1 | 0.02 | 0.89 | 1.00 | 1.12 | 8.14 | -18.33 | 20.56 |
| (Partial vs mean) | 1 | 0.80 | 0.37 | 0.75 | 11.56 | 12.92 | -19.30 | 42.42 |
| (Year-round vs mean) | 1 | 2.79 | 0.09 | 0.26 | -12.67 | 7.58 | -30.78 | 5.43 |
